# A Realist Review of Hospital‐to‐Home Transitions With Multiple Long‐Term Conditions Including Dementia

**DOI:** 10.1002/alz70858_097609

**Published:** 2025-12-24

**Authors:** Lauren Lawson, Matthew Cooper, Clare Tolley, Annette Hand, Hamde Nazar

**Affiliations:** ^1^ Newcastle University, Newcastle Upon Tyne, Tyne and Wear, United Kingdom; ^2^ Northumbria University, Newcastle, Tyne and Wear, United Kingdom

## Abstract

**Background:**

Hospital‐to‐home transitions are a complex process involving multiple providers for older adults (65+) with dementia, who often live with additional conditions. These transitions can compromise patient safety by increasing the risk of errors, miscommunication, and treatment delays, potentially leading to higher mortality, morbidity, and preventable readmissions. This realist review aimed to synthesise existing literature to develop a framework explaining how, for whom, and to what extent hospital‐to‐home transitions work for this population.

**Method:**

The review was pre‐registered on PROSPERO (CRD42023494003). Nine electronic databases (CINAHL/HMIC/Embase/MEDLINE/PQDT/PsycINFO/PubMed/Scopus/Web of Science) were systematically searched using key terms. Eligible documents focused on hospital‐to‐home transitions for older adults with any dementia and at least one other long‐term condition, living in the community, and/or caregivers, health, or social care professionals involved in their care. Interactions between contexts, mechanisms, and outcomes influencing transitions were synthesised to create the framework.

**Result:**

From inclusion of 68 peer‐reviewed and 2 grey literature documents, a framework for hospital‐to‐home transitions, with five dementia‐specific subcomponents was developed: dementia care management, knowledge, standards, system, and the role of family/friends. Caregivers experienced increased burden and emotional distress as multiple long‐term conditions including dementia was associated with greater responsibilities during transitions, particularly when patients had unmanaged conditions or behavioural or psychological symptoms of dementia. Healthcare professionals in numerous areas lacked appropriate dementia knowledge and training, which limited their ability to tailor discharge planning and manage care needs effectively. Without a standardised approach to recording and reporting dementia diagnoses, information transfer between providers was inadequate, hindering care continuity and effective discharge planning. Insufficient collaboration between health and social care providers and fragmented care pathways resulted in delays, unsafe discharges and increased reliance on caregivers, which exacerbated service gaps. Caregivers were often expected to perform additional medical and caring tasks in the community without training, which contributed to stress, decision‐making conflicts and potential hospital readmissions.

**Conclusion:**

Hospital‐to‐home transitions are a complex process with no simple solution. Our framework highlights the need for system‐level changes and tailored interventions to address population‐specific challenges, aiming to improve care continuity, enhance patient safety and support all those involved in post‐discharge care.

